# 氨基/五氟苯基双功能化磁性三组分共价有机骨架的制备及其在氟苯甲酸类物质吸附中的应用

**DOI:** 10.3724/SP.J.1123.2024.05024

**Published:** 2024-12-08

**Authors:** Yuelin WANG, Qian LIN, Jingnan GAO, Jiwei SHEN, Yinmao WEI, Chaozhan WANG

**Affiliations:** 合成与天然功能分子教育部重点实验室, 西北大学化学与材料科学学院, 陕西 西安 710127; Key Laboratory of Synthetic and Natural Functional Molecule of the Ministry of Education, College of Chemistry & Materials Science, Northwestern University, Xi’an 710127, China

**Keywords:** 多元策略, 三组分共价有机骨架, 磁性固相萃取, 合成后修饰, 氟苯甲酸, 高效液相色谱, multivariate strategy, tricomponent covalent organic framework, magnetic solid-phase extraction, post synthesis modification, fluorobenzoic acids, high performance liquid chromatography

## Abstract

氟苯甲酸类物质(FBAs)是合成许多药物、农用化学品和其他有机化合物的重要中间体,同时也被用作水示踪剂。FBAs作为地下水示踪剂具有环境友好、不会在地层中自然存在、性质稳定、地层吸附量小、用量少以及种类多且彼此间无干扰等优点,因此FBAs作为示踪剂的应用不断扩大。在采用FBAs追踪地层水运动时,由于地层水基质复杂且FBAs浓度低,直接分析较为困难,因此对复杂基质中FBAs进行样品预处理十分重要。本研究采用多元(MTV)策略,以2,4,6-三甲酰基间苯三酚(Tp)、3,3'-二羟基联苯胺(DHB)、3,3'-二硝基联苯胺(DNB)为构筑基元,通过希夫碱反应制备了一系列不同羟基/硝基比例的三组分共价有机骨架(COF)[Tp-DHB*_x_*DNB_(1-_*_x_*_)_] (*x*=0、0.25、0.5、0.75、1)。再通过合成后修饰,得到氨基/五氟苯基双功能化COF,即氟亲和/阴离子交换/反相混合模式吸附剂COF-PFB*_x_*-NH_2(1-_*_x_*_)_。通过考察COF-PFB*_x_*-NH_2(1-_*_x_*_)_对FBAs的吸附性能,筛选出COF-PFB_0.5_-NH_2(0.5)_用于FBAs的吸附。为了便于将材料从溶液中分离,以Fe_3_O_4_纳米粒子为核,采用原位生长法制备了氨基/五氟苯基双功能化磁性三组分共价有机骨架吸附剂(Fe_3_O_4_@COF-PFB_0.5_-NH_2(0.5)_),通过扫描电子显微镜、X射线光电子能谱及傅里叶变换红外光谱等表征,证明了该材料的成功制备。Fe_3_O_4_@COF-PFB_0.5_-NH_2(0.5)_对4-氟苯甲酸(4-FBA)、2,3,4-三氟苯甲酸(2,3,4-TFBA)、2,3,4,5-四氟苯甲酸(2,3,4,5-Tetra-FBA)和3,5-双三氟甲基苯甲酸(3,5-BTFMA)的最大吸附容量分别为73.5、64.9、38.4和253 mg/g。重复使用5次后,其吸附效果保持良好。在最佳吸附条件下,Fe_3_O_4_@COF-PFB_0.5_-NH_2(0.5)_对模拟地层水中4-FBA、2,3,4-TFBA、2,3,4,5-Tetra-FBA和3,5-BTFMA的吸附率分别为85.7%、86.5%、94.9%和82.4%,表明Fe_3_O_4_@COF-PFB_0.5_-NH_2(0.5)_在实际样品中FBAs的分离富集方面具有很大的应用潜力。

## Abstract (en)

Fluorobenzoic acids (FBAs) are important intermediates in the synthesis of many drugs, agrochemicals, and other organic compounds, and are also used as water tracers. FBAs are environmentally friendly, unnatural, have stable properties, are poorly adsorbed in aquifers, and are used in low dosages as groundwater tracers, which are their main advantages along with non-interfering diversity; as a result, their applications scope continues to expand. While FBAs are used to trace the movement of groundwater, direct analysis is often challenging owing to their low concentrations and the complexity of the groundwater matrix. Therefore, sample pretreatment for trace and ultra-trace FBAs in complex matrices is important prior to instrumental analysis. Covalent organic frameworks (COFs) are porous crystalline polymers constructed from organic units through strong covalent bonds and are characterized by low densities, large specific surface areas, permanent porosities, excellent chemical/thermal stabilities, and ease of functionalization for required purposes.

In this study, we used a multivariate (MTV) strategy to construct a series of tricomponent COFs with different hydroxy/nitro ratios from 2,4,6-trihydroxybenzene-1,3,5-tricarbaldehyde (Tp), 3,3'-dihydroxybenzidine (DHB), and 3,3'-dinitrobenzidine (DNB) as building blocks using Schiff-base reactions; these COFs are referred to as Tp-DHB*_x_*DNB_(1-_*_x_*_)_ (*x*=0, 0.25, 0.5, 0.75, and 1). We then anchored pentafluorophenyl and amino groups to these COFs via post-synthetic modification; the adsorption performance of the COF-PFB*_x_*-NH_2(1-_*_x_*_)_ samples prepared in this manner led to the selection of COF-PFB_0.5_-NH_2(0.5)_ for its FBAs-adsorbing ability. A magnetic fluorophilic/anion-exchange/reversed-phase mixed-mode COF adsorbent (Fe_3_O_4_@COF-PFB_0.5_-NH_2(0.5)_) was prepared via an in-situ growth method using Fe_3_O_4_ nanoparticles as cores to facilitate separation of the material from solution. Notably, the Fe_3_O_4_@COF-PFB_0.5_-NH_2(0.5)_ particles were very dispersible in water owing to their abundant amino groups and facilitated magnetic solid-liquid separation. In addition, Fe_3_O_4_@COF-PFB_0.5_-NH_2(0.5)_ exhibited excellent operational stability for the magnetic solid-phase extraction (MSPE) of FBAs in aqueous samples. The structural properties of Fe_3_O_4_@COF-PFB_0.5_-NH_2(0.5)_ were characterized using various techniques, including scanning electron microscopy (SEM), X-ray photoelectron spectroscopy (XPS), powder X-ray diffractometry (PXRD), Fourier-transform infrared spectroscopy, and the Brunauer-Emmett-Teller method, all of which revealed that Fe_3_O_4_@COF-PFB_0.5_-NH_2(0.5)_ had been successfully prepared; it also has uniformly distributed mesopores and a specific surface area of 107 m^2^/g. Adsorption-mechanism studies revealed that Fe_3_O_4_@COF-PFB_0.5_-NH_2(0.5)_ is highly adsorptive toward all four FBAs (4-fluorobenzoic acid (4-FBA), 2,3,4-trifluorobenzoic acid (2,3,4-TFBA), 2,3,4,5-tetrafluorobenzoic acid (2,3,4,5-tetra-FBA) and 3,5-bis(trifluoromethyl)benzoic acid (3,5-BTFMA)) through electrostatic, hydrogen bonding, F-F affinity, and *π-π* and hydrophobic interactions through synergy between the dual-functional fluorine and carboxyl binding sites, as well as the benzene rings in their skeletons. Isothermal FBAs-adsorption experiments involving Fe_3_O_4_@COF-PFB_0.5_-NH_2(0.5)_ revealed maximum adsorption capacities of 73.5, 64.9, 38.4, and 253 mg/g for 4-FBA, 2,3,4-TFBA, 2,3,4,5-tetra-FBA and 3,5-BTFMA, respectively. Adsorption kinetics were also investigated, which revealed that FBAs-adsorption by Fe_3_O_4_@COF-PFB_0.5_-NH_2(0.5)_ follows pseudo-second-order kinetics. Remarkably, no significant decrease in adsorption efficiency was observed after five adsorption-desorption cycles, highlighting good material reusability. We next optimized the factors that affect MSPE efficiency. Fe_3_O_4_@COF-PFB_0.5_-NH_2(0.5)_ exhibited adsorption efficiencies of 85.7%, 86.5%, 94.9%, and 82.4% for 4-FBA, 2,3,4-TFBA, 2,3,4,5-tetra-FBA and 3,5-BTFMA from simulated underground water, respectively, under the optimal extraction conditions, which indicates that Fe_3_O_4_@COF-PFB_0.5_-NH_2(0.5)_ holds significant potential for the separation and enrichment of FBAs in real samples.

氟苯甲酸类物质(FBAs)是合成抗菌药物的重要中间体,同时也被用作水示踪剂。FBAs作为地下水示踪剂的应用始于20世纪80年代^[1]^,其主要优点是环境友好、不会在地层中自然存在、性质稳定、地层吸附量小、用量少以及种类多且彼此间无干扰,可以同时在同一储层或位置进行多次研究而不受干扰^[2⇓-4]^,因此其作为示踪剂的应用领域不断扩大,被用作储层水示踪剂、地下水示踪剂、油藏技术示踪剂,同时也被用作碳封存技术中的泄漏指示剂^[5⇓⇓⇓⇓-10]^。在采用FBAs追踪地层水运动时,由于地层水中FBAs的浓度通常很低,且地层水基质复杂,直接分析较为困难,因此在仪器分析之前,对复杂基质中痕量和超痕量的FBAs进行样品预处理十分重要。

常用的痕量氟苯甲酸类化合物的分析方法有气相色谱-质谱联用法(GC-MS)^[11]^、离子色谱法^[12]^、超高效液相色谱法^[13]^和高效液相色谱法(HPLC)^[14]^。离子色谱法方便快速,但灵敏度较差,且易受实际水样中无机盐离子的干扰^[13]^。GC-MS检测灵敏度高,但样品预处理繁琐,衍生化反应条件严苛,且有副产物产生,影响准确性,检测周期较长,不易操作^[15]^。超高效液相色谱可高效分离,高灵敏度定量,预处理操作要求低,但仪器设备昂贵,不易推广^[16]^。高效液相色谱法可直接定量检测氟苯甲酸类物质,并可同时分析多种氟苯甲酸类物质,测定速度快,同时比GC-MS操作简便,比离子色谱法灵敏度高。水体中FBAs含量低,所以通常在进行高效液相色谱分析前,采用固相萃取(SPE)将微量的FBAs富集后进行检测。Müller等^[17]^基于二乙烯苯和*N*-乙烯基吡咯烷酮聚合物固相萃取及衍生化技术,开发了超痕量分析高盐度地层水样品中21种FBAs的气相色谱-质谱联用分析方法。Kubica等^[18]^采用C18为固定相的SPE方法,可以直接从富盐水域中同时高效富集19种FBAs。Kumar等^[19]^采用UiO-66-NH_2_为吸附剂的分散固相萃取(dSPE)结合GC-MS对23种痕量氟化芳香族羧酸进行了分析。以上研究表明固相萃取技术是痕量FBAs富集的有效方法,但现有吸附剂主要通过疏水作用、静电作用和氢键作用等吸附FBAs,缺乏选择性作用力,因而吸附选择性较差。因此引入选择性作用力以提高吸附剂选择性,对于FBAs的高效分离富集具有重要意义。

在吸附剂中引入氟元素,利用氟-氟亲和作用可有效提高对含氟小分子的吸附选择性,据此建立的氟固相萃取(F-SPE)已被用于选择性萃取含氟化合物。共价有机骨架(COF)结晶性较高,稳定性较好,可根据需求在骨架上引入特定的预置基团,使其具备功能上的可调控性等优点,近年来在样品前处理领域得到了广泛的应用^[20,21]^。通过在骨架中引入官能团,可以显著提高COF材料的吸附选择性和效率。将氟元素引入COF材料中,可以制备氟功能化的COF材料^[22⇓-24]^。目前,已有多种含氟COF吸附材料被应用于氟固相萃取。Zhang等^[25]^报道了一种简单的室温合成管状磁性氟化共价有机骨架(MCNT@TAPB-TFTA),用于高效富集环境样品中的超痕量多溴联苯醚,氟原子的引入改善了骨架的疏水性和对多溴联苯醚的吸附能力。然而,由于F-SPE的作用力主要依靠氟-氟亲和作用,目前的研究较为有限,而通过引入新的官能团以增加目标物和吸附剂之间的作用力,是提高吸附效果的一种可行途径。近年来出现了采用多元(multivariate, MTV)策略制备多官能团功能化COF的方法^[26⇓⇓-29]^。当两个或更多节点单体与一个以上的链接单元反应时,多种功能基团可以被整合到单个COF材料的骨架中,实现了利用3种或3种以上的构建单元来制备具有更多结构和性能多样性的COF。多元COF已在气体吸附^[26]^、催化^[30]^、储能^[31]^和发光^[32]^等方面显示出优势。Han等^[33]^使用“多元COF后修饰”策略开发了一系列混合链接离子COF,所得到的iCOF-AB-50表现出优异的碘吸附能力。Alsudairy等^[34]^提出了一种简便的微波辅助混合链接策略来制备多元COF吸附剂,该吸附剂对放射性碘蒸气表现出可调和超高的吸附性能。因此,多元COF提供了一个新的平台,大大扩展了多孔有机材料的结构和功能的设计性^[35,36]^。通过分析FBAs分子结构,可以发现FBAs分子中都含有氟原子和羧基,基于此特点,可以制备同时含有氟和氨基的多元COF,利用多元COF中的氟结构单元与FBAs分子中的氟原子之间的氟-氟亲和作用以及多元COF中的氨基与FBAs分子中羧基之间的静电作用,协同COF骨架中苯环与FBAs分子中苯环和疏水基团间的疏水和*π-π*作用,有望提高FBAs的吸附效率。

本研究采用多元策略,以2,4,6-三甲酰基间苯三酚(Tp)、3,3'-二羟基联苯胺(DHB)、3,3'-二硝基联苯胺(DNB)为构筑基元,制备了一系列用于吸附FBAs的三组分COF,通过改变链接单体中DHB/DNB的比例再结合后修饰来调节COF孔壁内五氟苯基与氨基的比例,得到一系列氟亲和/阴离子交换/反相混合模式吸附剂(COF-PFB*_x_*-NH_2(1-_*_x_*_)_)。考察了该系列吸附剂对FBAs的吸附性能,以Fe_3_O_4_为磁核,采用原位生长法得到氨基/五氟苯基双功能化磁性三组分COF吸附剂(Fe_3_O_4_@COF-PFB_0.5_-NH_2(0.5)_),探究了该材料的吸附等温线、吸附动力学、再生性能、吸附机理以及对模拟地层水中FBAs的吸附性能。

## 1 实验部分

### 1.1 仪器与试剂

TENSOR27傅里叶红外光谱仪(FI-IR,德国Bruker公司); D8ADVANCE X射线粉末衍射仪(XRD,德国Bruker公司); Tristar Ⅱ 3020表面吸附仪(BET,美国Micromeritics公司); H-600透射电子显微镜(TEM)和SV8010扫描电子显微镜(SEM)(日本日立公司); MPMS-ML-7超导量子干涉磁测量系统(SQUID,美国量子设计公司); LC-20A高效液相色谱仪(配备紫外检测器,日本岛津公司); STA 449C综合热分析仪(TGA,德国耐驰公司); PHI5000VersaProbeⅢ X射线光电子能谱(XPS,日本ULVAC-PHI公司)。

DNB(纯度≥97%)购于河南阿尔法化工有限公司;DHB(纯度≥99%)、2,3,4,5-四氟苯甲酸(2,3,4,5-Tetra-FBA,纯度≥98%)、2,3,4-三氟苯甲酸(2,3,4-TFBA,纯度≥98%)、4-氟苯甲酸(4-FBA,纯度≥98%)、五氟苯甲酰氯(PFB,纯度≥99%)、SnCl_2_(纯度97%)均购于麦克林生化科技有限公司;3,5-双三氟甲基苯甲酸(3,5-BTFMA,纯度≥98%)、2,3,4-三氟苯甲醛(2,3,4-TRA,纯度≥98%)、2,3,4-三氟苯胺(2,3,4-TRH,纯度≥98%)、4-甲基苯甲酸(*p*-TOA,纯度≥99%)、4-乙基苯酚(4-EPH,纯度≥97%)、均三甲苯均购于阿拉丁生化科技股份有限公司;乙腈(色谱纯)购于康科德科技有限公司;四氢呋喃、冰醋酸、盐酸、三乙胺、1,4-二氧六环、丙酮、二氯甲烷、甲醇、丙酮均购于天津科茂化学试剂有限公司。

### 1.2 色谱条件

C18色谱柱(150 mm×4.6 mm, 5 μm, Agilent),流动相A为乙腈,流动相B为10 mmol/L NaH_2_PO_4_ (pH=3.0)。分析4-FBA、2,3,4-TFBA和2,3,4,5-Tetra-FBA时,流动相A和B的比例为7∶3 (v/v);分析3,5-BTFMA时,流动相A和B的比例为52∶48(v/v)。流速均为1.0 mL/min,进样量为20 μL,柱温30 ℃。检测波长223 nm。

### 1.3 COF-PFB_0.5_-NH_2(0.5)_的制备

#### 1.3.1 三组分共价有机骨架(Tp-DHB*_x_*DNB_(1-_*_x_*_)_)的制备

参考Huang等^[37]^的方法合成Tp。称取Tp (0.0504 g, 0.230 mmol)和总量0.340 mmol但不同比例的DHB和DNB于聚四氟乙烯内衬管中,DHB的物质的量分数为*x* (*x*=0、0.25、0.5、0.75、1), DNB的物质的量分数为1-*x*,再加入均三甲苯(3 mL)、1,4-二氧六环(3 mL)和3 mol/L HAc (1 mL),将混合物超声10 min使其分散均匀,然后将其放入反应釜中,于120 ℃加热反应72 h,得到红棕色沉淀。依次用丙酮、四氢呋喃、二氯甲烷和丙酮洗涤多次,得到产物Tp-DHB*_x_*DNB_(1-_*_x_*_)_。

#### 1.3.2 五氟苯基功能化三组分共价有机骨架(COF-PFB*_x_*)的合成

称取Tp-DHB*_x_*DNB_(1-_*_x_*_)_ (100 mg)和三乙胺(2*x* mmol)于烧瓶中,加入二氯甲烷(4 mL),将烧瓶放置于冰浴中。然后将PFB(2*x* mmol)和二氯甲烷(1 mL)混合均匀后滴加到烧瓶中,室温下反应3 h。反应结束后,用水和甲醇交替洗涤产物,于50 ℃真空干燥,得到产物COF-PFB*_x_*。

#### 1.3.3 COF-PFB*_x_*-NH_2(1-_*_x_*_)_的合成

将COF-PFB*_x_*和SnCl_2_ (8 g)加入含无水四氢呋喃(25 mL)的烧瓶中,超声10 min。将混合物在75 ℃下回流6 h, 10000 r/min下离心10 min得到红棕色固体。将固体用1 mol/L盐酸、蒸馏水、丙酮洗涤多次。以甲醇为溶剂,将产物索氏提取24 h, 50 ℃真空干燥。COF-PFB*_x_*-NH_2(1-_*_x_*_)_的合成路线如图1所示。

**图 1:**
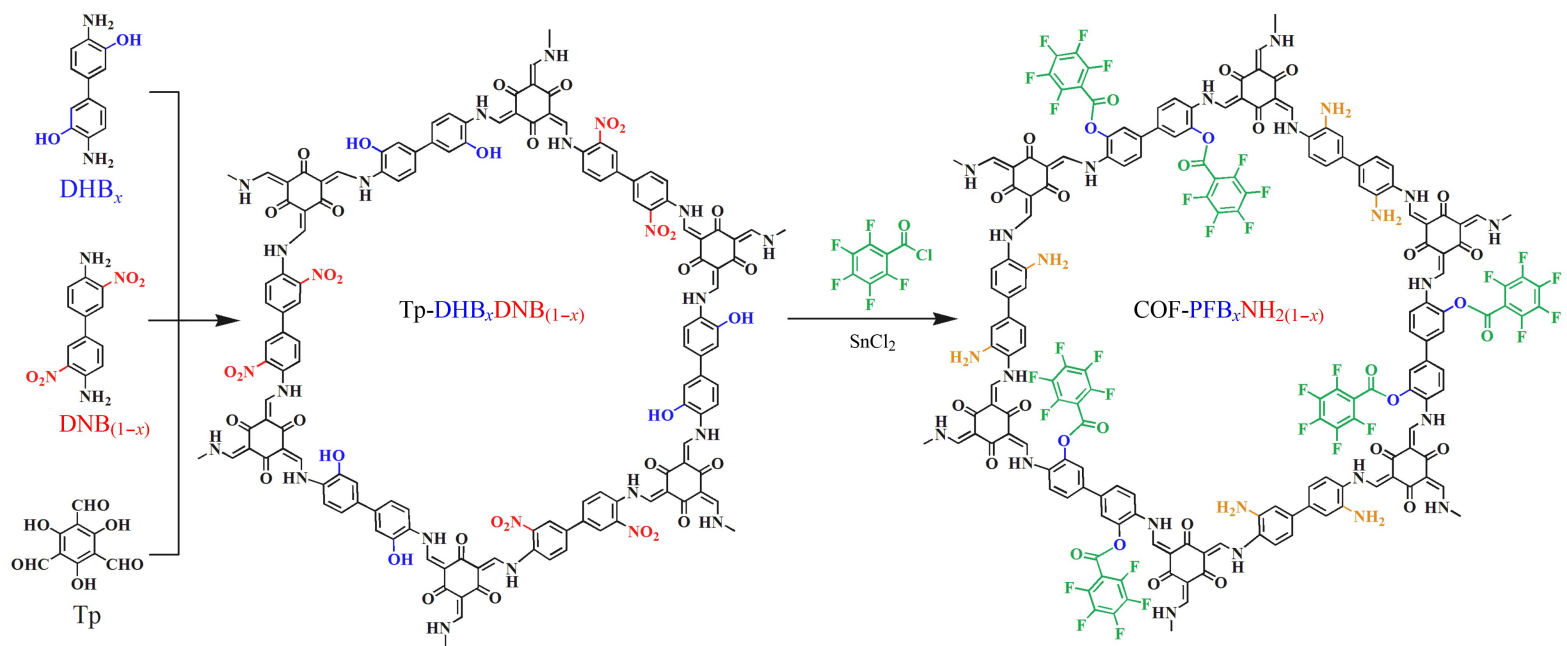
MTV策略衍生的COF-PFB*_x_*-NH_2(1-_*_x_*_)_合成示意图

### 1.4 Fe_3_O_4_@COF-PFB_0.5_-NH_2(0.5)_的制备

#### 1.4.1 磁性三组分共价有机骨架(Fe_3_O_4_@COF)的合成

采用原位生长法^[38]^,在Fe_3_O_4_表面生长COF。参考Wang等^[39]^的方法合成Fe_3_O_4_-Tp。称取Fe_3_O_4_-Tp(0.1g)、Tp(0.0504 g, 0.230 mmol)、DHB(0.0368 g, 0.500 mmol)和DNB(0.0466 g, 0.500 mmol)于聚四氟乙烯内衬管中,再加入均三甲苯(3 mL)、1,4-二氧六环(3 mL)和3 mol/L HAc(1 mL),后续实验步骤与1.3.1节相同,得到产物Fe_3_O_4_@COF。

#### 1.4.2 五氟苯基功能化磁性三组分共价有机骨架(Fe_3_O_4_@COF-PFB_0.5_)的合成

取900 mg Fe_3_O_4_@COF、三乙胺(94.0 μL, 1.00 mmol)和二氯甲烷(4 mL)于烧瓶中,将烧瓶置于冰浴中。然后将PFB(92.0 μL, 1.00 mmol)和二氯甲烷1 mL混合均匀后滴加到烧瓶中,其余操作同1.3.2节,得到Fe_3_O_4_@COF-PFB_0.5_。

#### 1.4.3 Fe_3_O_4_@COF-PFB_0.5_-NH_2(0.5)_的合成

按照1.3.3节步骤合成Fe_3_O_4_@COF-PFB_0.5_-NH_2(0.5)_,通过外部磁场分离并洗涤,得到产物Fe_3_O_4_@COF-PFB_0.5_-NH_2(0.5)_。

### 1.5 FBAs吸附实验

用甲醇分别配制1 mg/mL 4-FBA、2,3,4-TFBA、2,3,4,5-Tetra-FBA和3,5-BTFMA的储备液,再以超纯水稀释至0.2 mg/L。准确称量5 mg Fe_3_O_4_@COF-PFB_0.5_-NH_2(0.5)_于离心管中,分别加入2 mL 0.2 mg/L的分析物溶液,25 ℃下振荡10 min。磁分离后取上清液并用微孔滤膜(0.22 μm)过滤,用超纯水淋洗粒子3次后,采用1 mL醋酸-甲醇(1∶1, v/v)洗脱(25 ℃, 60 min),磁分离后收集洗脱液并用微孔滤膜(0.22 μm)过滤,然后用HPLC-UV分别检测母液、上清液和洗脱液中FBAs的浓度。根据式(1)~(3)分别计算吸附率(*A*)、吸附容量(*Q*, mg/g)和回收率(*R*)。所有实验结果均为3次平行实验结果的平均值。


(1) $$A=\frac{C_{0}-C_{e}}{C_{0}}\times 100\%$$

(2) $$Q=\frac{C_{0}-C_{e}}{m}\times V_{0}$$

(3) $$R=\frac{C_{i}V_{i}}{C_{0}V_{0}}\times 100\%$$

式中,*C*_0_、*C*_e_和*C*_i_ (mg/L)分别为溶液中FBAs初始质量浓度、平衡质量浓度和洗脱液质量浓度,*V*_0_和*V*_i_ (mL)分别为初始溶液和洗脱液的体积,*m* (mg)为吸附剂的质量。

### 1.6 Fe_3_O_4_@COF-PFB_0.5_-NH_2(0.5)_吸附FBAs机理研究

为了探究分析物与目标吸附剂的相互作用机理,设计了4组实验分别探究静电作用、氢键作用、氟-氟亲和作用和疏水作用。分别将5 mg吸附剂加入2 mL 4组分析物与干扰物质量浓度均为20 mg/L的混合溶液中,在25 ℃下吸附60 min。第一组:2,3,4-TFBA和2,3,4-TRA;第二组:2,3,4-TFBA与2,3,4-TRH;第三组:2,3,4-TFBA和*p*-TOA;第四组:2,3,4-TFBA和4-EPH。附表1中列出了所涉及分析物的结构性质(www.chrom-China.com)。

### 1.7 Fe_3_O_4_@COF-PFB_0.5_-NH_2(0.5)_用于吸附模拟地层水中的FBAs

采用模拟地层水作为样品基质(组成如表1),探究Fe_3_O_4_@COF-PFB_0.5_-NH_2(0.5)_的实际应用潜力。取5 mg吸附剂加入2 mL含0.2 mg/L 4种FBAs的模拟地层水中,于室温25 ℃下振荡10 min。在磁铁辅助下分离吸附剂和上清液,上清液经滤膜(0.22 μm)过滤后进行HPLC-UV检测,并根据式(1)计算吸附率。

**表 1:** 模拟地层水的组成

Ingredient	C/(mg/L)	Ingredient	C/(mg/L)
NaCl	36.85	SrCl_2_·6H_2_O	0.4
KCl	0.63	NaHCO_3_	0.2
CaCl_2_·2H_2_O	3.8	Na_2_SO_4_	0.05
MgCl_2_·6H_2_O	2.55	Crude oil	10^*^
BaCl_2_·2H_2_O	0.1		

* mL/L.

## 2 结果与讨论

### 2.1 基于MTV策略的氨基/五氟苯基双功能化三组分COF材料的制备

由于FBAs的结构含有苯基、氟基和羧基,我们设计了一种氨基/五氟苯基双功能化三组分COF吸附剂,即氟亲和/阴离子交换/反相混合模式吸附剂,用于提高FBAs的吸附选择性和吸附效率。首先利用Tp与DHB/DNB作为混合功能单体,通过席夫碱反应制备三组分COF吸附剂,再通过后修饰将五氟苯基和氨基引入COF,研究五氟苯基和氨基的比例对吸附效果的影响,结果如图2所示。

**图 2:**
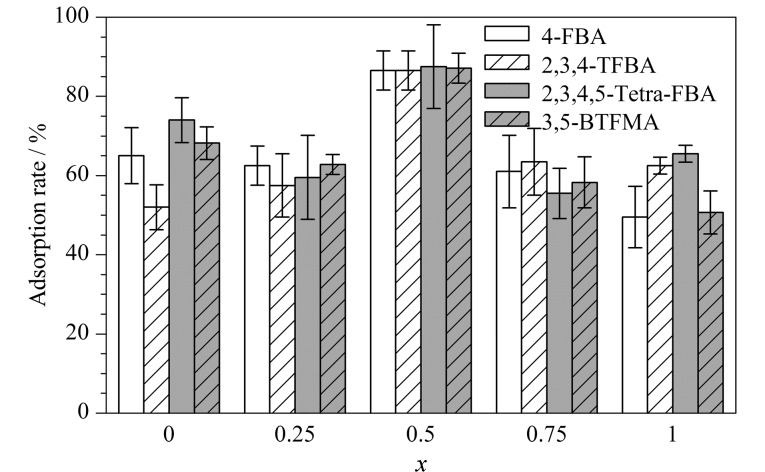
COF-PFB*_x_*-NH_2(1-_*_x_*_)_对4种FBAs的吸附率(*n*=3)

结果表明,COF-PFB_0.5_-NH_2(0.5)_吸附FBAs的效果最好,对4种分析物(相关参数见附表2)的吸附率均能达到86%以上,这归因于吸附过程中吸附剂上五氟苯基与分析物之间的氟-氟亲和作用、氨基与羧基之间的静电作用,以及COF骨架中苯环与FBAs分子中苯环和疏水基团间的疏水和*π-π*作用等多种作用力的协同效果。为了便于快速简单地将吸附剂从溶液中分离出来,以Fe_3_O_4_纳米粒子为核,采用原位生长法制备了Fe_3_O_4_@COF-PFB_0.5_-NH_2(0.5)_,后续均采用该材料进行实验。

### 2.2 Fe_3_O_4_@COF-PFB_0.5_-NH_2(0.5)_的表征

通过扫描电镜和透射电镜对材料进行表征。如图3a和3b所示,Fe_3_O_4_@SiO_2_是表面粗糙的球形纳米粒子。从图3c和3d可以看出,包覆上COF层后,Fe_3_O_4_@COF-PFB_0.5_-NH_2(0.5)_最终呈现出具有Fe_3_O_4_核和COF外壳的核壳结构。COF包覆在Fe_3_O_4_@SiO_2_表面,表现为表面较光滑的核壳结构且壳层厚度不均匀,核壳结构的纳米粒子外相连的是COF交织形成的折叠片层,表明了Fe_3_O_4_@COF-PFB_0.5_-NH_2(0.5)_的制备成功。

**图 3:**
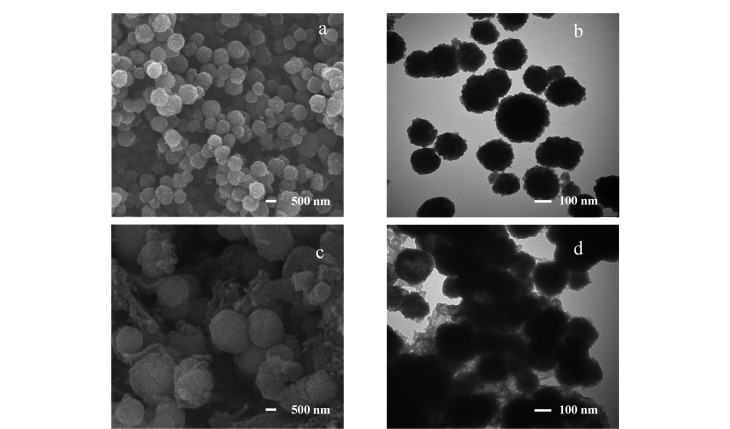
Fe_3_O_4_@SiO_2_与Fe_3_O_4_@COF-PFB_0.5_-NH_2(0.5)_的SEM和TEM图

对制备的材料进行X射线光电子能谱表征和元素含量分析,结果如图4a和表2所示。Fe_3_O_4_@SiO_2_在结合能为102、285、532和711 eV处出现峰值,分别对应Si 2*p*、C 1*s*、O 1*s*和Fe 2*p*。包覆COF层后的Fe_3_O_4_@COF在400 eV处观察到N 1*s*的吸收峰(图4b),通过氟修饰后在687 eV出现F 1*s*的吸收峰(图4c)。根据表2可知F元素含量占1.33%,进一步说明五氟苯基的成功修饰。将COF侧链的硝基还原后得到了Fe_3_O_4_@COF-PFB_0.5_-NH_2(0.5)_,其光谱分析显示Fe、O、Si、C、N和F的存在。Fe_3_O_4_@COF-PFB_0.5_-NH_2(0.5)_与Fe_3_O_4_@COF相比氧含量下降0.43%,表明硝基成功还原为氨基。以上结果表明Fe_3_O_4_@COF-PFB_0.5_-NH_2(0.5)_成功合成。

**图 4:**
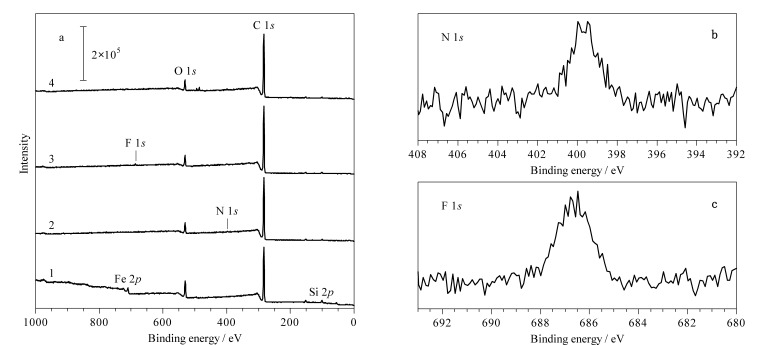
(a)不同纳米粒子的XPS全谱图和Fe_3_O_4_@COF-PFB_0.5_-NH_2(0.5)_的(b)N 1*s*谱图、(c) F 1*s*谱图

**表 2:** 不同磁性纳米粒子的元素含量

Material	Elemental contents/%					
	C 1s	O 1s	Si 2p	Fe 2p	N 1s	F 1s
Fe_3_O_4_@SiO_2_	59.7	25.3	13.5	1.41	-	-
Fe_3_O_4_@COF	87.4	8.06	2.47	0.37	1.68	-
Fe_3_O_4_@COF-PFB_0.5_	86.0	7.43	2.89	0.28	2.05	1.33
Fe_3_O_4_@COF-PFB_0.5_-NH_2(0.5)_	86.8	7.63	2.40	0.19	2.51	0.47

图5a为磁性纳米粒子的FT-IR表征图。在584 cm^-1^处的吸收峰归因于Fe-O-Fe伸缩振动,Fe_3_O_4_@SiO_2_在1084、961和792 cm^-1^处的吸收峰分别归因于Si-O-Si不对称、Si-O对称和Si-O-Si对称伸缩振动^[40]^。Fe_3_O_4_@COF在1583 cm^-1^出现苯环骨架中C=N的伸缩振动峰,在795 cm^-1^处为芳环的C-H弯曲振动峰,在1335 cm^-1^和1511 cm^-1^存在硝基的对称和不对称伸缩振动峰。Fe_3_O_4_@COF-PFB_0.5_在1010 cm^-1^出现C-O伸缩振动,表明五氟苯基修饰成功^[41]^。Fe_3_O_4_@COF-PFB_0.5-_NH_2(0.5)_在1211 cm^-1^出现伯胺的C-N伸缩振动峰,表明硝基还原为氨基。以上结果表明COF壳层是通过希夫碱反应形成的^[42]^,并已成功包覆在Fe_3_O_4_纳米颗粒表面。采用XRD图谱分析了合成材料的晶型结构(图5b), 30.1°、35.4°、43.1°、57.0°和62.6°处的衍射峰分别指向Fe_3_O_4_晶体的(220)、(311)、(400)、(422)、(511)和(440)晶面^[43]^,合成的复合材料的衍射峰位置没有变化,表明包覆COF后磁性粒子的晶型未改变。2*θ*=1.4°的衍射峰(图5b插图)归属于COF壳层。以上结果表明Fe_3_O_4_@COF-PFB_0.5_-NH_2(0.5)_的成功制备。在77 K下采用氮吸附-解吸附实验评价所制备的COF的孔结构(图5c)。Fe_3_O_4_@COF-PFB_0.5_-NH_2(0.5)_的吸附曲线具有典型的Ⅳ型氮吸附等温线特征,表明其具有介孔特性。材料比表面积为107 m^2^/g,从图5c中插图可看出孔径分布较窄,孔径集中分布在1.7~2.3 nm,孔体积为0.214 cm^3^/g,表明所制备的COF磁性材料具有永久的孔隙率、狭窄的孔径分布和较大的比表面积,有利于提高吸附性能。

**图 5:**
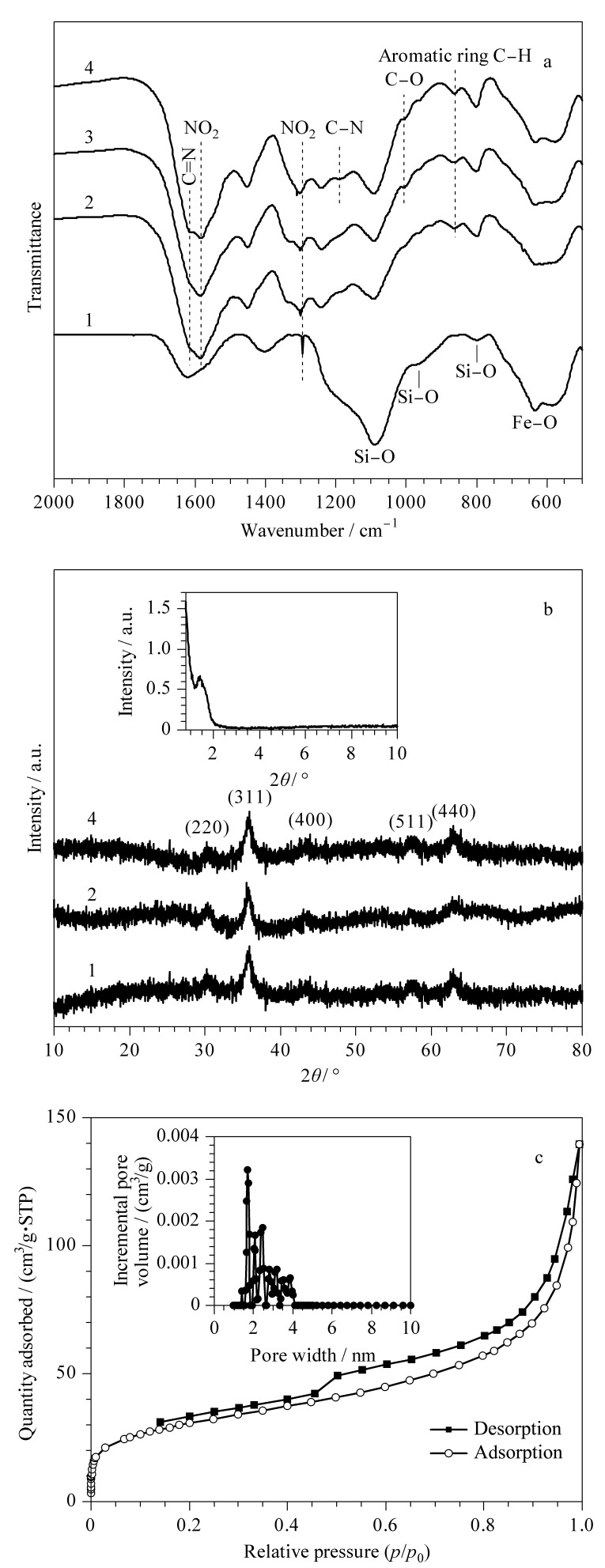
材料的(a)FT-IR、(b)XRD和(c)氮吸附-解吸附分析

### 2.3 FBAs吸附条件优化

#### 2.3.1 盐浓度

在固相萃取过程中,离子强度可能会影响萃取结果。采用质量分数为0~10%的NaCl评估盐浓度对萃取的影响。如图6a所示,在盐浓度为0时,Fe_3_O_4_@COF-PFB_0.5_-NH_2(0.5)_对FBAs的吸附率均在80%以上;当盐浓度增加至2%时,对4-FBA、2,3,4-TFBA、2,3,4,5-Tetra-FBA和3,5-BTFMA的吸附率分别降低至70.0%、40.4%、48.4%和50.3%,说明盐浓度对FBAs的吸附具有较大影响,并且对2,3,4-TFBA、2,3,4,5-Tetra-FBA和3,5-BTFMA的影响更大。这是由于离子强度的增加会导致吸附剂与分析物之间的静电作用减弱^[44]^,且吸附剂的活性位点会被Cl^-^占据^[43]^,导致吸附效率降低。2,3,4-TFBA和2,3,4,5-Tetra-FBA的吸附率在盐浓度增加至5%时,出现了降低,在盐浓度为5%时降低到最小值,在盐浓度为5%~10%时略有回升。而4-FBA和3,5-BTFMA在盐浓度为2%以后,吸附率逐渐升高,可以归因于盐浓度增加使得FBAs的溶解度降低,并使疏水相互作用增强^[45]^。综上所述,4种分析物的吸附率在盐浓度为0时最高,因此后续实验均采用不加盐的方式进行。

**图 6:**
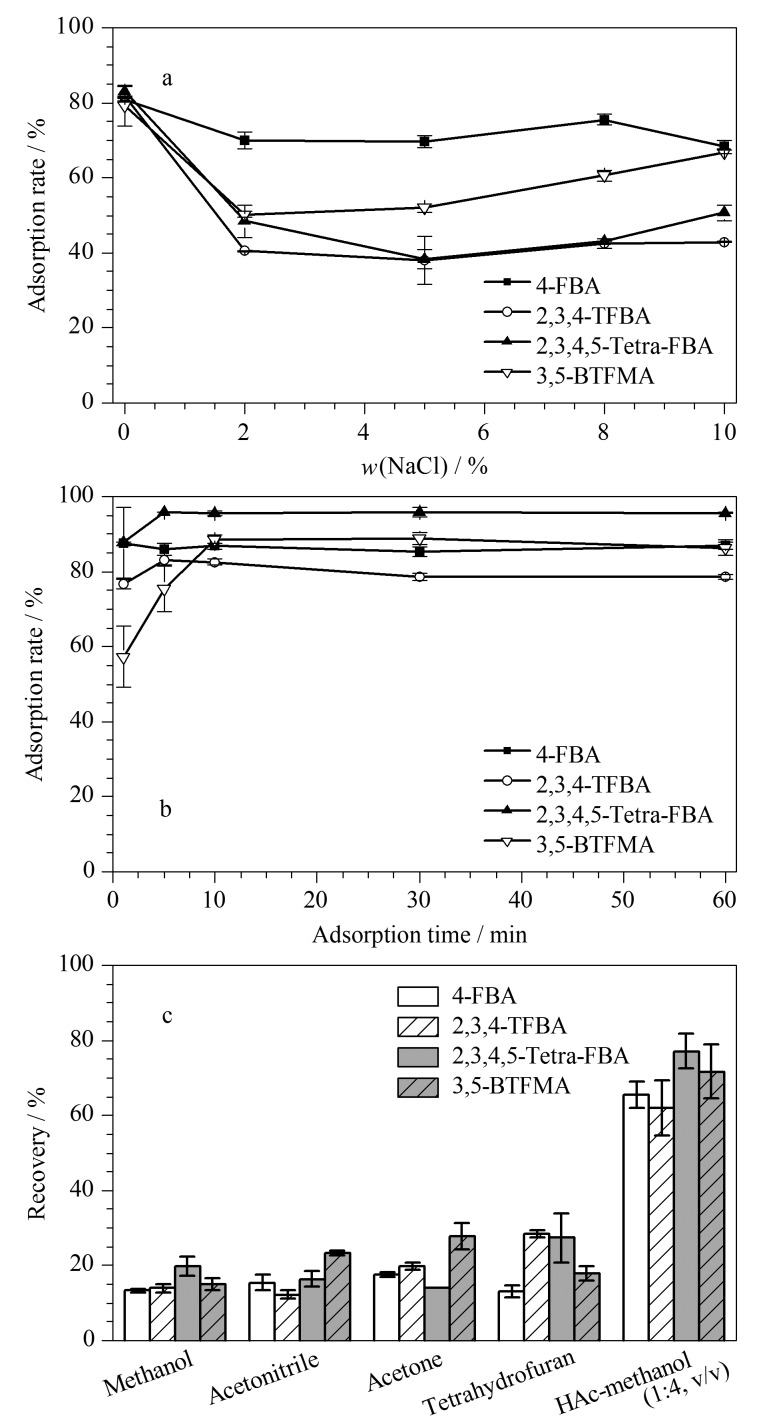
(a)盐浓度、(b)吸附时间、(c)洗脱剂类型对FBAs吸附的影响(*n*=3)

#### 2.3.2 吸附时间

吸附时间是萃取过程中的重要因素^[46]^,实验在0~60 min内评估了吸附时间对萃取的影响。如图6b所示,4种FBAs在短时间内均表现出较好地去除效果。在0~5 min时4种FBAs的吸附率均随时间延长迅速提高。2,3,4-TFBA和2,3,4,5-Tetra-FBA的吸附率在5 min时分别达到83.2%和95.7%,此后保持不变。而4-FBA与3,5-BTFMA的吸附在10 min时达到平衡,此后无明显提高。综上所述,为保证FBAs均达到吸附平衡,选择10 min为最佳吸附时间。

#### 2.3.3 洗脱剂类型

洗脱剂通过破坏吸附剂与分析物之间的吸附作用来达到洗脱效果,合适的洗脱剂可以提高萃取回收率。本研究中吸附剂对FBAs的吸附作用主要依靠氟-氟亲和、疏水相互作用、静电相互作用和氢键作用,因此在解吸过程中,可以考虑使用含酸的有机溶剂破坏相关吸附作用力以实现解吸。如图6c所示,分别选择甲醇、乙腈、丙酮、四氢呋喃和醋酸-甲醇(1∶4, v/v)作为洗脱溶剂。以甲醇、乙腈、丙酮、四氢呋喃作为洗脱剂时,回收率均处于10%~30%。在甲醇中引入醋酸后,由于酸性条件下FBAs以分子形态存在,静电相互作用被破坏^[47]^, 4-FBA、2,3,4-TFBA、2,3,4,5-Tetra-FBA和3,5-BTFMA的回收率分别增加到65.5%、61.9%、77.1%和71.7%。因此,选择醋酸-甲醇(1∶4, v/v)为洗脱剂。

### 2.4 吸附等温线

在298 K下选取初始质量浓度为5~200 mg/L的4-FBA、2,3,4-TFBA、2,3,4,5-TFBA和3,5-BTFMA进行吸附实验,考察等温吸附行为。吸附量与FBAs初始浓度的关系如附图1所示,随着FBAs浓度的增加,吸附量逐渐增加并最终达到吸附平衡,这主要是由于吸附剂的活性位点逐渐饱和。如附图2所示,采用Langmuir和Freundlich模型拟合,拟合结果如附表3所示,Langmuir对4-FBA、2,3,4-TFBA、2,3,4,5-TFBA和3,5-BTFMA的吸附数据拟合后的决定系数(*R*^2^)分别为0.9176、0.9755、0.9932和0.9877,而Freundlich模型拟合后的*R*^2^分别为0.9032、0.9528、0.9574和0.9494,均小于Langmuir模型拟合的*R*^2^。因此,Fe_3_O_4_@COF-PFB_0.5_-NH_2(0.5)_对FBAs的吸附更符合Langmuir模型,表明FBAs以单层形式吸附在Fe_3_O_4_@COF-PFB_0.5_-NH_2(0.5)_上。4-FBA、2,3,4-TFBA、2,3,4,5-TFBA和3,5-BTFMA的最大吸附容量分别为73.5、64.9、38.4和253 mg/g。通过Freundlich线性方程计算得出1/*n*<1,说明吸附主要为化学过程。

### 2.5 吸附动力学

通过测定吸附剂在1~60 min内对FBAs的吸附量来研究吸附动力学。如附图3所示,在吸附初期材料表面的吸附位点较多,吸附速率较快^[48]^。随着吸附进行,吸附位点逐渐饱和,吸附容量基本保持不变,吸附达到平衡。采用伪一阶和伪二阶动力学模型来探究FBAs在Fe_3_O_4_@COF-PFB_0.5_-NH_2(0.5)_上的吸附动力学,拟合结果如附表4所示。4-FBA、2,3,4-TFBA、2,3,4,5-TFBA和3,5-BTFMA的伪二阶动力学拟合的*R*^2^分别为0.9980、0.9987、0.9995和0.9987,远大于伪一阶动力学拟合的*R*^2^,说明伪二阶动力学模型能更好地描述吸附过程,表明化学吸附是控制速率的主要因素。

### 2.6 合成重复性和重复利用性

在相同实验条件下用3个批次的Fe_3_O_4_@COF-PFB_0.5_-NH_2(0.5)_吸附FBAs,如图7a所示,结果表明不同批次吸附剂的吸附效果差异小,说明该吸附剂具有良好的合成重复性。同时,对其进行吸附-解吸附循环实验,发现经过5次循环使用后吸附剂对4种FBAs的吸附率无明显降低(图7b),表明该吸附剂具有良好的重复利用性。

**图 7:**
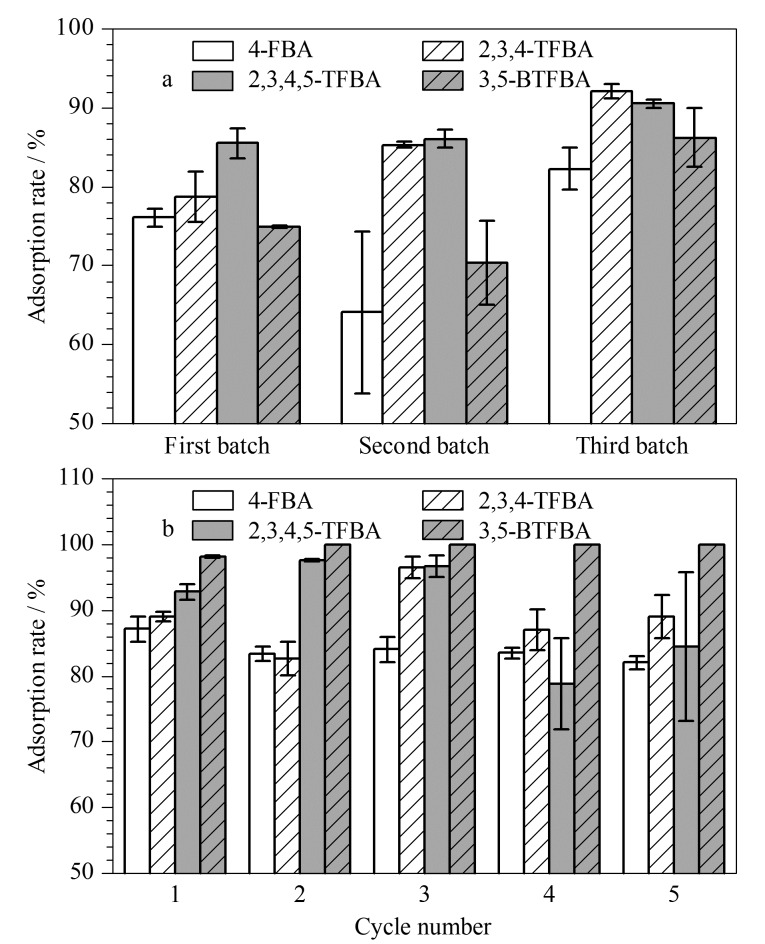
吸附剂的(a)合成重复性和(b)重复利用性(*n*=3)

### 2.7 吸附机理

#### 2.7.1 静电作用和氢键作用

通过对比Fe_3_O_4_@COF-PFB_0.5_-NH_2(0.5)_对2,3,4-TFBA与2,3,4-TRA或2,3,4-TRH的混合溶液吸附率的差异,探究静电作用和氢键作用的影响(见图8 Group 1和2)。结果显示,吸附剂对2,3,4-TFBA吸附率高于2,3,4-TRA和2,3,4-TRH,与2,3,4-TRA和2,3,4-TRH相比,2,3,4-TFBA带有羧基,能与吸附剂所带氨基之间产生静电相互作用和氢键作用^[49]^,表明吸附过程中存在静电作用力和氢键作用力。

**图 8:**
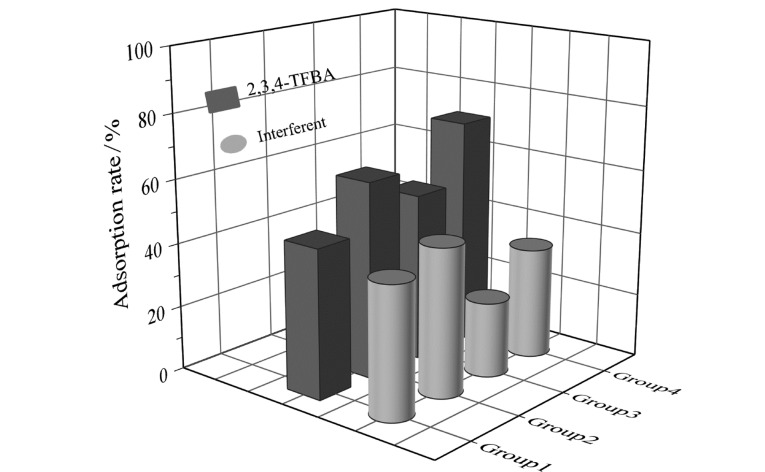
吸附剂对4组物质的吸附效率对比

#### 2.7.2 氟-氟作用

*p*-TOA与2,3,4-TFBA相比只具有羧基,不具有三氟基团,通过Fe_3_O_4_@COF-PFB_0.5_-NH_2(0.5)_对*p*-TOA和2,3,4-TFBA混合溶液的吸附实验探究吸附过程中的氟-氟作用(见图8 Group 3)。结果表明,吸附剂对2,3,4-TFBA的吸附率高于*p*-TOA,吸附剂上带有五氟苯基,能与2,3,4-TFBA所带的氟基产生氟-氟亲和作用^[50]^,表明吸附剂与目标分析物之间存在氟-氟作用力。吸附剂对*p*-TOA也存在一定的吸附效果,归因于其与吸附剂之间存在静电相互作用、氢键和疏水相互作用。

#### 2.7.3 疏水作用

探究了Fe_3_O_4_@COF-PFB_0.5_-NH_2(0.5)_对2,3,4-TFBA和4-EPH的吸附效果(见图8 Group 4)。结果表明,吸附剂对2,3,4-TFBA的吸附率远高于4-EPH。4-EPH与2,3,4-TFBA的log *K*_ow_相近,但不含有氟基、羧基,因此不能与吸附剂之间产生氟-氟亲和作用、静电相互作用及氢键作用,但由于苯环的存在使4-EPH能够与吸附剂之间产生疏水相互作用,所以对4-EPH也存在一定的吸附效果,证实了疏水相互作用在吸附中的作用。

综上所述,Fe_3_O_4_@COF-PFB_0.5_-NH_2(0.5)_与FBAs之间的吸附涉及静电作用、氢键作用、氟-氟亲和作用和疏水作用等多重作用。

### 2.8 Fe_3_O_4_@COF-PFB_0.5_-NH_2(0.5)_用于模拟地层水中FBAs的吸附

地层水是指油藏边部和底部的边水和底水、层间水以及与原油同层的束缚水的总称。地层水在地层中长期与岩石和原油接触,通常含有相当多的金属盐类,如钾盐、钠盐、钙盐、镁盐等,尤其以钾盐、钠盐最多。为进一步考察Fe_3_O_4_@COF-PFB_0.5_-NH_2(0.5)_对实际样品中复杂基质的抗干扰能力,以模拟地层水为样品,探究Fe_3_O_4_@COF-PFB_0.5_-NH_2(0.5)_对模拟地层水中FBAs的吸附效果。本研究考察了Fe_3_O_4_@COF-PFB_0.5_-NH_2(0.5)_对纯净水和模拟地层水中4种FBAs的吸附效果,结果如表3所示。模拟地层水中4-FBA、2,3,4-TFBA、2,3,4,5-Tetra-FBA和3,5-BTFMA的吸附率分别为85.7%、86.5%、94.9%和82.4%,与其在纯净水中的吸附效果相比,吸附率无明显变化,表明Fe_3_O_4_@COF-PFB_0.5_-NH_2(0.5)_在吸附地层水中FBAs方面具有很大的应用潜力。

**表 3:** Fe_3_O_4_@COF-PFB_0.5_-NH_2(0.5)_对纯净水和模拟地层水中FBAs的吸附效果(*n*=3)

Analyte	Pure water			Simulated formation water	
	Adsorption rate/%	RSD/ %		Adsorption rate/%	RSD/ %
4-FBA	84.5	0.4		85.7	3.1
2,3,4-TFBA	94.2	4.9		86.5	2.1
2,3,4,5-Tetra-FBA	97.4	4.1		94.9	0.5
3,5-BTFMA	85.4	3.6		82.4	0.3

## 3 结论

采用MTV策略结合合成后修饰制备了一系列用于FBAs吸附的氨基/五氟苯基双功能化三组分COF(COF-PFB*_x_*-NH_2(1-_*_x_*_)_),即氟亲和/阴离子交换/反相混合模式吸附剂。其中,COF-PFB_0.5_-NH_2(0.5)_对FBAs的吸附效果最好。并以Fe_3_O_4_纳米粒子为核,采用原位生长法制备了磁性氟亲和/阴离子交换/反相混合模式COF吸附剂(Fe_3_O_4_@COF-PFB_0.5_-NH_2(0.5)_),将该材料用于吸附水中的氟苯甲酸类物质,在最佳条件下对4种物质均有较高的吸附容量,较快的吸附效率和较好的重复利用性。因此,Fe_3_O_4_@COF-PFB_0.5_-NH_2(0.5)_是一种有前途的吸附材料,为地层水等复杂基质中FBAs的富集提供了新思路,在样品前处理中存在一定的应用潜力,并扩展了共价有机骨架材料的应用。但是该吸附剂结晶度较低,比表面积也不高,同时没有用于实际地层水样中FBAs的分析。在以后的工作中,应进一步改善材料的结晶度,更充分地研究吸附选择性和竞争吸附情况,并将吸附剂应用于实际水体中FBAs的分离富集。
